# Optimized Isolation and Cryopreservation of Functional Mitochondria for Transplantation and Therapeutic Applications

**DOI:** 10.3390/cells15141279

**Published:** 2026-07-16

**Authors:** Vikky Awasthi, Aasthika Das, Meriem Bkhache, Abeer Alshambky, Glenn S. Gerhard, Karim Bahmed, Timothy Cashman, Rihab Bouchareb

**Affiliations:** 1 Center for Metabolic Disease Research (CMDR), Department of Cardiovascular Sciences, Lewis Katz School of Medicine (LKSM), Temple University, 3500 North Broad Street, Philadelphia, PA 19140, USA; fnu.vikky@temple.edu (V.A.); aasthika.das@temple.edu (A.D.); mebkhache@gmail.com (M.B.); abeer.alshambky@temple.edu (A.A.); 2 Department of Medical Genetics and Molecular Biochemistry, Temple University, 3500 North Broad Street, Philadelphia, PA 19140, USA; glenn.gerhard@tuhs.temple.edu; 3 Center for Inflammation and Lung Research, Department of Cardiovascular Sciences, Lewis Katz School of Medicine at Temple University, Philadelphia, PA 19140, USA; karim.bahmed@temple.edu; 4 Temple Heart & Vascular Institute, Lewis Katz School of Medicine at Temple University, Philadelphia, PA 19140, USA; timothy.cashman@tuhs.temple.edu

**Keywords:** mitochondria isolation, mitochondria transplantation, cryopreservation of mitochondria

## Abstract

**Highlights:**

**What are the main findings?**

- Efficient mitochondrial isolation that preserves post-transplantation viability and function.
- Development of a cryopreservation strategy for mitochondria.

**What are the implications of the main findings?**

- Methodological implication: a fast, reproducible isolation workflow that preserves mitochondrial bioenergetics and dynamics can standardize mitochondrial transplantation experiments and reduce variability driven by isolation-related damage.
- Translational/biobanking implication: demonstrating functional recovery after low-temperature storage supports the feasibility of pre-prepared, quality-controlled mitochondrial stocks (“organelle biobanks”), enabling scalable studies and improving logistics for future therapeutic development.

**Abstract:**

Mitochondria play a central role in numerous physiological and pathological processes, and mitochondrial transplantation is emerging as a promising strategy to restore cellular function and mitigate disease. The success of this approach depends critically on the methods used to isolate, preserve, and retrieve intact, functional mitochondria. **Objective**: To optimize an isolation strategy that preserves mitochondrial integrity, dynamics, and metabolic activity and to evaluate conditions that enable short-term storage for future organelle biobanking applications. **Methods**: We compared a mitochondria isolation method developed in our laboratory (Protocol A) with a commercially available kit (Protocol B). Donor mitochondria were isolated from proximal tubular cells and transplanted into HEK293T recipient cells. Mitochondrial functionality was assessed following transfer into HEK293T cells by measuring reactive oxygen species (MitoSOX Red), oxygen consumption rate (OCR) using Seahorse XF analysis, and high-resolution imaging of mitochondrial morphology and dynamics. We further evaluated mitochondrial storage at low temperature and subsequent functional recovery. **Results**: Protocol A enabled faster isolation (~30 min) than Protocol B (~80 min) and yielded mitochondria with higher transplantation efficiency, greater OCR, preserved dynamic morphology, and lower oxidative stress. Mitochondria isolated using Protocol A remained metabolically active after transplantation and continued to exhibit fission and fusion, whereas those isolated using Protocol B showed reduced dynamic behavior. Importantly, mitochondria isolated with Protocol A retained functional integrity after low-temperature storage, supporting their potential for standardized preservation. **Conclusions**: This study presents a robust, efficient, and reproducible isolation and frozen-storage protocol that yields highly functional mitochondria suitable for transplantation. The ability to preserve mitochondrial function after storage further highlights the potential for developing organelle biobanks to support future research and therapeutic applications.

## 1. Introduction

Mitochondria are double-membraned, semi-autonomous organelles that possess their own genome and exhibit dynamic behavior. While classically recognized as the cell’s primary energy-generating organelle due to their role in ATP production via oxidative phosphorylation, mitochondria also serve as central hubs for a wide range of metabolic and signaling processes that are critical for cellular adaptation, differentiation, and survival [1,2]. They play a pivotal role in regulating intracellular calcium homeostasis, particularly through close physical and functional interactions with the sarcoplasmic/endoplasmic reticulum (SR/ER), facilitating calcium buffering and signaling [3,4]. Furthermore, mitochondria modulate cell fate by releasing key tricarboxylic acid (TCA) cycle metabolites, such as citrate, α-ketoglutarate, and succinate, which influence epigenetic modifications, immune responses, and cellular proliferation [3,4,5]. TCA cycle metabolites, primarily considered as byproducts of cellular metabolism, are important for the biosynthesis of macromolecules such as nucleotides [5,6], lipids [7], and proteins [8]. Besides cellular homeostasis, mitochondria play a pivotal role in cellular differentiation [9] and cell death by regulating apoptosis [10], autophagy [11], and reactive oxygen species (ROS) generation [12]. As a primary center for cellular metabolism, mitochondria are vulnerable to dysfunction, which may arise through damage to mitochondrial DNA or mitochondrial proteins. Mitochondrial dysfunction has been implicated in a variety of common causes of morbidity and mortality, including cardiovascular disease [13], renal disease [14], neurodegeneration [15], metabolic syndrome [16], and cancer [17].

Recently, mitochondrial transplantation has been investigated as a potential therapeutic strategy to address mitochondrial dysfunction [18], in which healthy mitochondria can be isolated and transplanted into cells to restore mitochondrial function, reduce cellular oxidative stress, and promote cell survival by counteracting apoptosis [19,20]. Mitochondria can be isolated from different tissues, particularly the liver and cultured kidney cells, using several methods [21]. These methods primarily involve cell disruption, where the mitochondrial fraction can be obtained through differential or gradient centrifugation.

Differential centrifugation separates cellular components based on their sedimentation rates at different centrifugation speeds. This method offers an efficient approach, involving tissue disruption via homogenization, followed by low-speed centrifugation to remove cell debris, and a subsequent high-speed centrifugation step to collect mitochondria [22]. The homogenization step is crucial, as the force required to disrupt cells while preserving mitochondrial membranes varies between tissue types. A critical factor influencing the quality and quantity of isolated mitochondria is the variation in cell type and cell disruption methods. Cell disruption methods may be enzymatic or mechanical (often using a Dounce homogenizer). The enzymatic method is limited by incubation time, enzyme activity, and cost. Furthermore, processing multiple samples simultaneously makes it increasingly challenging and time-consuming. Disruption may also be carried out using detergents [23]. However, excessive detergent incubation can disrupt mitochondrial integrity, resulting in nonfunctional organelles.

Following tissue disruption and cell lysis, mitochondria can be separated from cellular debris using differential or density-gradient centrifugation. Density-gradient centrifugation employs sucrose or Percoll gradients, allowing fractions containing distinct cellular components to be collected after high-speed centrifugation (often ultracentrifugation [24]). Although ultracentrifugation yields a highly pure mitochondrial fraction, it is time-consuming and requires access to an ultracentrifuge. Moreover, the isolated mitochondria must be washed to remove residual sucrose or Percoll before transplantation. Alternative approaches include mitochondrial isolation using magnetic beads conjugated to outer mitochondrial membrane markers, such as TOM20 [25]. This method yields a highly pure mitochondrial fraction in a short time; however, its scalability and cost are limitations. Another important factor in isolation methodologies is the composition of the isolation buffers, as it can directly influence mitochondrial viability and function. These buffers must preserve mitochondria in a native, functional state suitable for transplantation.

In this study, we describe a method we developed to isolate mitochondria from cells and compare two isolation approaches. This aspect is critical for enabling the development of organelle biobanking, in which isolated mitochondria can be preserved, cataloged, and readily deployed for downstream applications. Establishing robust storage conditions that maintain mitochondrial integrity and bioenergetic function provides a foundation for standardized mitochondrial repositories for both research and therapeutic use. Such organelle biobanks would enhance reproducibility across laboratories, support large-scale studies requiring consistent mitochondrial preparations, and accelerate the clinical translation of mitochondrial transplantation and metabolic therapies.

Overall, our protocol is simple, reproducible, and user-friendly, and it preserves mitochondrial dynamics, bioenergetic function, and transplantation efficiency.

## 2. Materials and Methods

### 2.1. Cell Culture

Primary tubular epithelial cells were isolated from the kidneys of transgenic mice expressing mitochondria-targeted Dendra2, a green-to-red photoswitchable monomeric fluorescent protein, and were used in this study. Dendra2 mice were obtained from The Jackson Laboratory. Upon exposure to 405 nm laser light, Dendra2 fluorescence irreversibly shifts from green to red, enabling quantitative assessment of mitochondrial fusion and transport dynamics [26,27,28]. Photoconversion was used to perform ratiometric and time-resolved fluorescence analyses, minimizing reliance on absolute fluorescence intensity.

Proximal tubular cells (donor cells) were cultured in DMEM/F12 medium supplemented with 5% fetal bovine serum (FBS) and 1% penicillin–streptomycin until reaching 70–80% confluence. These cells served as a source of fluorescent mitochondria for transplantation experiments. Cell viability and apoptosis (Fisher Scientific, Pittsburgh, PA, USA) were assessed using commercially available assays according to the manufacturer’s instructions.

HEK293T cells were cultured in DMEM (Fisher Scientific, Pittsburgh, PA, USA) supplemented with 10% FBS and used at 70–80% confluence for experiments evaluating mitochondrial transfer efficiency and live-cell imaging of mitochondrial dynamics.

### 2.2. Apoptosis Assay

Primary proximal tubular cells (PTCs) were cultured in DMEM/F-12 medium supplemented with 5% fetal bovine serum (FBS) until reaching 70–80% confluency. On the day of treatment, the culture medium was replaced, and cells were exposed to hydrogen peroxide (H_2_O_2_) at a final concentration of 600 µM for 18 h. Following treatment, cells were stained with Annexin V–APC (BD Pharmingen, San Diego, CA, USA #550475) and propidium iodide (PI; BD Pharmingen, #51-6211E) according to the manufacturer’s instructions. Fluorescent images were acquired using confocal microscopy. Apoptotic cells (Annexin V–positive) were quantified using ImageJ software, 1.50i, and normalized to the total number of cells. Statistical significance was determined using an unpaired *t*-test in GraphPad Prism5, with results expressed as mean ± SEM.

### 2.3. Mitochondria Isolation Protocol A

Proximal tubular cells were grown in 10-cm culture dishes to ~80% confluency. The cells were trypsinized, washed with isolation buffer A (210 mM mannitol, 70 mM sucrose, 10 mM HEPES, 1 mM EDTA), and centrifuged at 300× *g* for 5 min at 4 °C. The pellet was resuspended in 5 mL of ice-cold buffer A and centrifuged again. The resulting pellet was then resuspended in 1 mL of isolation buffer A. The suspension was homogenized by passing it 30 times through a 25-gauge needle. The homogenate was centrifuged at 2000× *g* for 5 min at 4 °C, and the pellet was discarded. The supernatant was then centrifuged at 13,000× *g* for 10 min at 4 °C. The resulting pellet was resuspended in PBS1X to measure protein concentration. 100 ug of protein was used per condition.

### 2.4. Mitochondria Isolation Protocol B

For Protocol B, we used a commercial Qiagen kit (Qproteome Mitochondria Isolation Kit, Germantown, MD, USA) and followed the manufacturer’s instructions. Briefly, cells were grown in 10-cm culture dishes to ~70% confluency. After trypsinization, the cell suspension was centrifuged at 300× *g* for 10 min at 4 °C, and the supernatant was carefully removed and discarded. The cells were then washed with 0.9% NaCl, resuspended in ice-cold lysis buffer, and supplemented with protease inhibitors. After incubation for 10 min at 4 °C, the suspension was centrifuged at 1000× *g* for 10 min at 4 °C. The pellet was resuspended in 1.5 mL of ice-cold disruption buffer, and mechanical disruption was aided by passing the suspension through a syringe 10 times. The lysate was centrifuged again at 1000× *g* for 10 min at 4 °C. The supernatant was transferred to a microcentrifuge tube and centrifuged at 6000× *g* for 10 min at 4 °C. The resulting pellet, containing mitochondria, was washed with 1 mL of mitochondria storage buffer and centrifuged at 6000× *g* for 20 min at 4 °C before being used for subsequent procedures.

### 2.5. Western Blotting

Mitochondria were isolated from proximal tubular cells using Protocols A and B. The protein concentration of the isolated mitochondria was determined using the BCA assay (Bio-Rad, Hercules, CA, USA). Mitochondrial proteins were extracted using RIPA lysis buffer (Santa Cruz Biotechnology, Dallas, TX, USA). Thirty micrograms of protein were loaded onto a 12% SDS-PAGE gel. Proteins were transferred to PVDF membranes using a Trans-Blot system (Bio-Rad) under default settings. Membranes were blocked in TBS-Tween containing 5% bovine serum albumin (BSA), incubated with primary antibodies overnight at 4 °C, washed three times for 5 min each, and then incubated with secondary antibodies (1:5000 dilution). Blots were developed using SuperSignal™ West Pico PLUS Chemiluminescent Substrate (Thermo Fisher Scientific, Germantown, MD, USA, 34580) and imaged with the iBright 1500 system. Primary antibodies used were TOM20 (Cell Signaling Technology, Danvers, MA, USA, 42406S), HDAC (Cell Signaling Technology, 34589T), BAX (Cell Signaling Technology, Danvers, MA, USA, 2772S), LAMP2 (Thermo Fisher Scientific, Germantown, MD, USA, PA1-654A), and GAPDH (Cell Signaling Technology, Danvers, MA, USA, 97166).

### 2.6. Immunostaining

Cells were cultured on 22-mm glass coverslips. Samples were fixed with 4% paraformaldehyde (PFA) and blocked for 1 h at room temperature in blocking buffer (PBS containing 5% normal serum and 0.3% Triton X-100). Cells were then incubated overnight at 4 °C with primary antibodies diluted in antibody dilution buffer (PBS containing 1% BSA and 0.3% Triton X-100) and washed three times. Cells were incubated with Phalloidin (Thermo Fisher Scientific, Germantown, MD, USA, A34055) for 20 min and with secondary antibodies for 2 h at room temperature. After washing with 1× TBS, coverslips were mounted using a mounting medium containing DAPI (Thermo Fisher Scientific, Germantown, MD, USA). Images were acquired using a Leica DMi8 confocal microscope. MitoSOX staining was performed according to the manufacturer’s protocol (Thermo Fisher Scientific, Germantown, MD, USA, M36008). Briefly, cells were incubated with 200 nM MitoSOX Red for 30 min at 37 °C in 5% CO_2_. Cells were then fixed and imaged using the Leica confocal microscope.

### 2.7. Measurement of Complex II (Succinate: Coenzyme Q Oxidoreductase) Activity SDH Assay

Assay for SDH activity was performed as described previously [29]. Purified mitochondria (0.1 mg of protein) were suspended in 50 mM (K) phosphate buffer (pH 7.4) containing 3 mM potassium ferricyanide (III) acting as an exogenous electron acceptor. A decrease in the absorption (420 nm) upon the addition of 50 mM succinate was measured to measure the rate of potassium ferrocyanide (II) formation at 30 °C for 2 min. The reaction rate was calculated as nmol ferrocyanide formed per minute per mg protein (ε_420_ for potassium ferricyanide = 1040 M^−1^ cm^−1^). SDH activity is used here as a representative indicator of mitochondrial enzymatic integrity [29].

### 2.8. Intramitochondrial Calcium Indicators Fura-4

The isolated mitochondria were subjected to Fura-4 dye (Invitrogen, Germantown, MD, USA, F14201), a calcium indicator with different excitation depending on the calcium-binding state. The fluorescence intensity ratio at these excitation wavelengths indicates intracellular calcium levels. Calcium concentration is obtained by using fluorescence detected at 509 nm.

### 2.9. Measurement of OCR

HEK293T cells (2 × 10^4^ cells/well) were seeded into collagen IV–coated 96-well Seahorse plates (Agilent Biosciences, Santa Clara, CA, USA). Isolated mitochondria were added to each well at 50 µg/well, and cells were incubated for 24 h. Oxygen consumption rate (OCR) was measured using a Seahorse XF-96 Analyzer (Agilent Technologies, North Billerica, MA, USA). The day before the assay, the sensor cartridge was hydrated overnight in the calibration buffer provided by Agilent. On the day of the experiment, the culture medium was removed, and the wells were washed once with Seahorse assay medium. Then, 180 µL of Seahorse assay medium was added to each well. Oligomycin, FCCP, and rotenone/antimycin A were loaded into ports A, B, and C, respectively, to achieve final well concentrations of 1.5 µM, 1 µM, and 0.5 µM. The standard Mito Stress Test protocol was used for OCR measurements.

### 2.10. Cryopreservation of Mitochondria

PTCs mitochondria isolated using Protocols A and B were resuspended in a DMSO-based recovery buffer at a final concentration of 1 mg/mL and stored at −80 °C. For transplantation experiments, cryopreserved mitochondria were thawed at 37 °C, washed once with respiration buffer to remove residual cryoprotectant, and resuspended in the same buffer before use.

### 2.11. Microscopic Analysis and Mitochondrial Transplantation

Isolated green, fluorescent mitochondria (from PTCs) (100 µg/well) were directly added to recipient HEK293T cells in culture medium for 24 h, then washed three times with PBS1X before adding fresh medium for 24 h. All imaging was performed using a Leica DMi8 confocal microscope at 48 h post-transplantation. As previously described for photoconversion analysis [1], Dendra2 in its unconverted (green) and photoconverted (red) states was excited using the 488 nm and 561 nm laser lines, respectively. Photoconversion was achieved by illuminating a defined region of interest with a 405 nm laser (4% power) for 59 bleaching iterations, with an exposure time of 5.14 s per iteration [26,27,28]. For live-cell imaging, a 63× objective was used to monitor mitochondrial fusion dynamics. A subset of mitochondria was photoconverted (red) and tracked over time using time-lapse imaging. Image analysis was performed using Fiji (ImageJ 1.5i) with the Mitochondria Analyzer plugin [2]. Three-dimensional reconstruction and analysis of mitochondrial networks were conducted using the Fiji 3D Viewer plugin.

### 2.12. Flow Cytometric Analysis of Mitochondrial Uptake

HEK293T cells were cultured and incubated with 100 µg of green-fluorescent mitochondria isolated using Protocols A and B. After 48 h, HEK293T cells (recipient) were trypsinized and washed twice with flow buffer (1× PBS supplemented with 1% fetal bovine serum) to remove non-internalized mitochondria. HEK293T cells were then resuspended in a final volume of 300 µL of flow buffer.

Samples were protected from light and analyzed using a BD FACS Discover flow cytometer. Untreated cells were used as a negative control to establish baseline fluorescence and define gating thresholds. Cells incubated with mitochondria isolated using Protocols A or B were analyzed using the same gating strategy to quantify Dendra2-positive cells. In addition, isolated green, fluorescent mitochondria alone were analyzed to define the signal corresponding to free (non-cell-associated) mitochondria and exclude debris or extracellular events during analysis.

### 2.13. Statistical Analyses

Statistical analyses were performed using GraphPad Prism (version 8). Differences between the two groups were assessed using two-tailed paired or unpaired Student’s *t*-tests, as appropriate. For comparisons among three or more groups, a one-way ANOVA was performed. Experiments were replicated more than 4 times.

*p* values were considered statistically significant as follows: * *p* < 0.05, ** *p* < 0.01, *** *p* < 0.005, and **** *p* < 0.001.

## 3. Results

### 3.1. Assessment of Mitochondrial Isolation Methods

**Figure 1:**
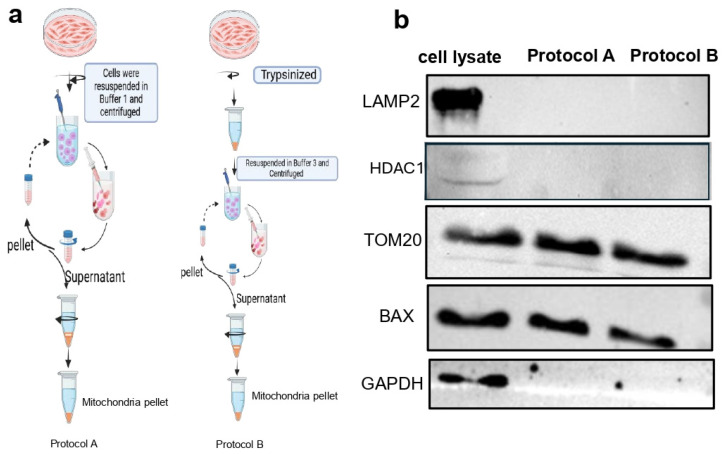
Comparative analysis of mitochondrial isolation using Protocols A and B. (**a**) Schematic overview illustrating the workflow for mitochondrial isolation using Protocols A and B. (**b**) Western blot analysis of isolated mitochondrial fractions probed with antibodies against GAPDH (cytosolic marker), BAX (mitochondrial outer membrane protein), TOM20 (mitochondrial marker), HDAC (nuclear marker), and LAMP2 (lysosomal marker) to assess purity and potential cross-contamination.

We compared mitochondrial isolation Protocols A and B using proximal tubular cells (PTCs) expressing a mitochondria-targeted, photoconvertible Dendra2 variant. This system enables real-time visualization of mitochondrial morphology, dynamics, and fate following transplantation through live-cell imaging and photoconversion approaches. Upon 405 nm illumination, Dendra2 irreversibly shifts from green to red, allowing tracking of mitochondrial fusion, transport, and integration within recipient cells [26]. Protocol A employed mechanical disruption using custom-formulated sucrose/mannitol buffers, whereas Protocol B relied on proprietary commercial buffers (Figure 1a). Mitochondria isolated using Protocol A were obtained more rapidly (<30 min) compared with those isolated using Protocol B. Cell viability was tested using Annexin V, as shown in Supplementary Figure S1. Western blotting was performed to assess the purity of mitochondria isolated using Protocols A (lab-developed process) and B (commercial kit) (Figure 1a). Mitochondria from both protocols were subjected to western blot analysis (Figure 1b), with whole-cell lysate serving as a control. Both Protocols A and B showed no detectable cytosolic (GAPDH), nuclear (HDAC), or lysosomal (LAMP2) contamination, indicating high mitochondrial purity. Additionally, mitochondria isolated by both protocols exhibited robust expression of the mitochondrial marker TOM20, confirming the successful enrichment of mitochondrial fractions (Figure 1b, Figure S4).

### 3.2. Efficiency of Mitochondrial Transplantation

To evaluate the functional efficiency of isolated mitochondria, we performed a transplantation assay. HEK293T cells (recipient) cultured in DMEM supplemented with 10% fetal bovine serum at ~70% confluence were incubated with 100 µg of mitochondria isolated from a tubular epithelial cell line expressing green-fluorescent mitochondria.

**Figure 2:**
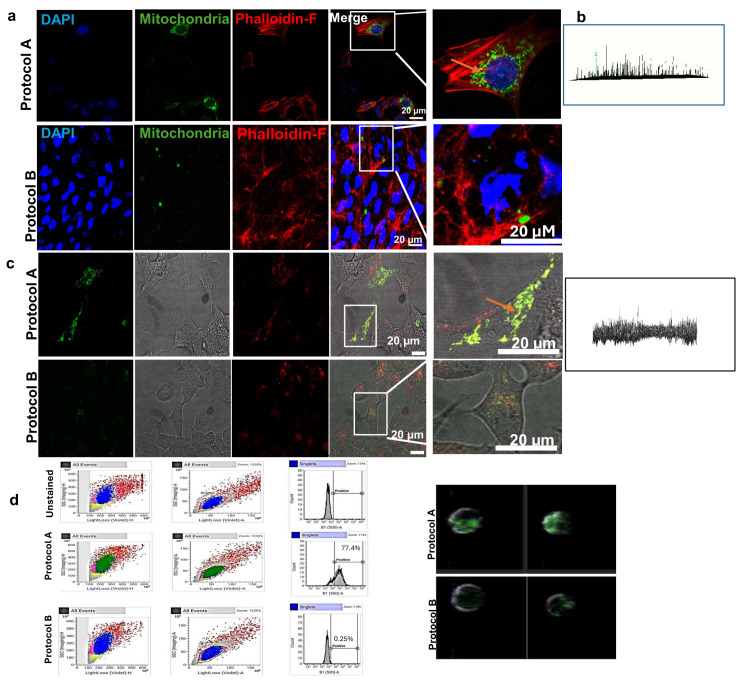
Efficiency of mitochondrial transfer into recipient cells. Mitochondria were isolated using Protocol A or Protocol B and quantified by a bicinchoninic acid (BCA) assay. A total of 100 µg of green-fluorescent mitochondria was added per well to recipient cells seeded in 6-well plates. (**a**) Immunofluorescence analysis using phalloidin-F (red) demonstrates enhanced mitochondrial uptake (orange arrows) and dynamic distribution in cells receiving mitochondria isolated with Protocol A compared to Protocol B. (**b**) Representative 3D surface rendering showing in the *Z-axis* cytoplasmic localization of mitochondria isolated with Protocol A upper panel and Protocol B lower panel. (**c**) Assessment of mitochondrial internalization and colocalization in vitro. Transplanted mitochondria (green) from Protocols A and B were compared with endogenous mitochondria labeled with Mito Tracker Red. Cells receiving mitochondria from Protocol A exhibit increased uptake and greater colocalization with the native mitochondrial network. The right panel shows a representative 3D surface rendering showing cytoplasmic localization of mitochondria isolated with Protocol A. (**d**) Flow cytometric quantification of cells positive for transplanted mitochondria (unstained cells used as control). Flow cytometry representative images (right) confirm efficient internalization of mitochondria isolated with Protocol A.

Confocal imaging revealed that mitochondria isolated using Protocol A exhibited an elongated, dynamic morphology and aligned along actin filaments (Figure 2a). In contrast, mitochondria isolated using Protocol B displayed a predominantly rounded morphology. To assess the spatial organization of transplanted mitochondria, a three-dimensional (3D) surface rendering was performed (Figure 2b, upper-right panel). Protocol A mitochondria formed dynamic intracellular networks, whereas Protocol B yielded no detectable signal.

To distinguish transplanted mitochondria from endogenous mitochondria, the recipient HEK293T cells were co-stained with Mito Tracker Red (Figure 2b). Time-lapse confocal imaging demonstrated a progressive increase in Dendra2 fluorescence, consistent with efficient mitochondrial uptake (Figure 2c). Green fluorescence corresponded to transplanted mitochondria, while red fluorescence represented endogenous mitochondrial networks (Supplementary Video S1). 3D surface reconstruction further confirmed their distinct spatial distribution (Figure 2c, right panel).

Flow cytometric analysis was used to quantify mitochondrial uptake efficiency. Mitochondria isolated using Protocol A were detected in 77.4% of recipient cells, whereas only 0.25% of cells were positive following incubation with Protocol B mitochondria (Figure 2d). Representative flow cytometry plots further confirmed the presence of green-fluorescent mitochondria within recipient cells (Figure 2d, right panel). The gating strategy is available in the supplement, Figure 2a,b.

### 3.3. Mitochondrial Morphology and Dynamics Post-Transplantation

Quantitative analysis of mitochondrial morphology was performed 48 h post-transplantation using the Mitochondria Analyzer plugin in Fiji (ImageJ 1.5i). This tool enables high-throughput measurement of multiple morphological parameters, including area, perimeter, form factor, aspect ratio, and branching.

**Figure 3:**
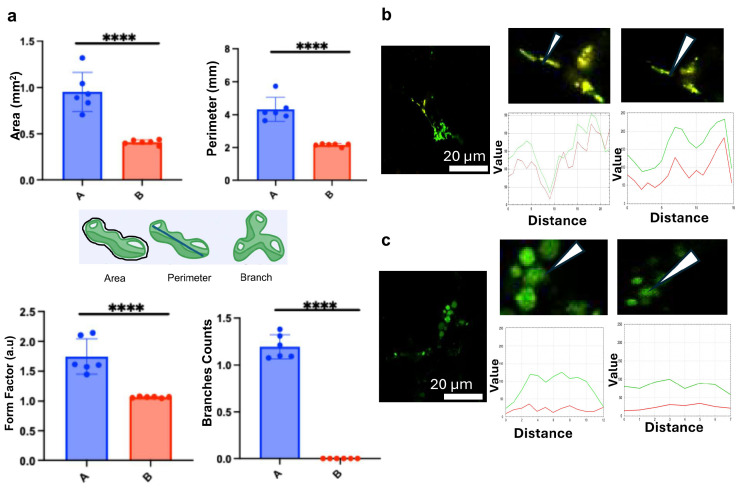
Mitochondrial network complexity and fusion dynamics following transplantation. Dendra2-labeled mitochondria isolated using Protocol A or Protocol B were transferred into recipient cells and analyzed after 48 h. (**a**) Quantitative morphometric analysis from 2D images (ImageJ Mitochondria Analyzer) shows increased mitochondrial area, perimeter, form factor, and branching in cells receiving Protocol A mitochondria, indicating enhanced network complexity. The schematic illustrates measured parameters. (**b**,**c**) Mitochondrial fusion dynamics assessed by Dendra2 photoconversion (405 nm) and time-lapse confocal microscopy. Representative sequences (white rectangle) for Protocol A (**b**) and Protocol B (**c**) show fusion events (white arrowheads. Data are mean ± S.E.M. Statistical significance was determined by an unpaired two-tailed Student’s *t*-test (**** *p* < 0.0001).

Mitochondria isolated using Protocol A exhibited significantly greater area and perimeter (Figure 3a, upper panels), indicating more extensive and elongated mitochondrial networks compared with Protocol B. In addition, form factor, calculated as perimeter^2^/(4π × area), an inverse measure of circularity in which higher values indicate greater elongation, and branch number (quantified using skeletonization and branch-counting algorithms) were significantly increased in mitochondria from Protocol A (Figure 3a, lower panels), reflecting enhanced mitochondrial interconnectivity and dynamic behavior.

To assess mitochondrial fusion, we leveraged the photoconvertible properties of Dendra2, which irreversibly switches fluorescence from green to red upon exposure to a 405 nm laser. Following photoconversion, live-cell time-lapse imaging was used to monitor red and green fluorescence signals over time [26,27,28]. Fusion events were identified by the mixing of red and green signals, indicative of matrix continuity and mitochondrial content exchange. Using this approach, fusion events were readily observed in mitochondria transplanted using Protocol A, confirming dynamic remodeling of the mitochondrial network (Figure 3b,c). In contrast, mitochondria isolated using Protocol B remained rounded and spatially isolated, with no evidence of fusion or fission, indicating limited post-transplant integration and functionality (Supplementary Video S2).

Together, these morphological and photoconversion-based analyses demonstrate that mitochondria isolated using Protocol A maintain structural integrity and dynamic behavior to a significantly greater extent than those isolated using Protocol B.

### 3.4. Bioenergetic and Functional Assessment of Transplanted Mitochondria

**Figure 4:**
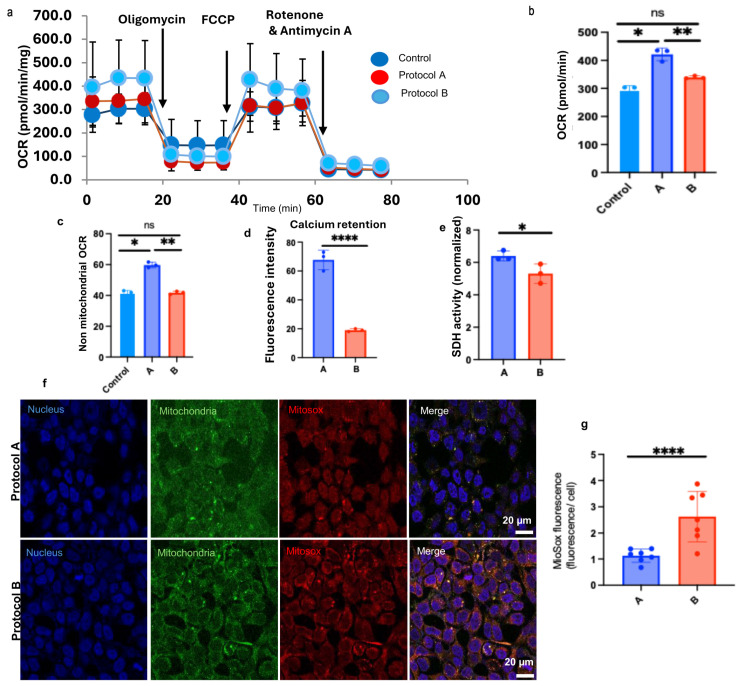
Bioenergetic and functional assessment of transplanted mitochondria. HEK293T cells (recipient) were incubated with 100 µg of green mitochondria isolated using Protocol A or Protocol B and analyzed 48 h post-transplantation. Cells without mitochondrial transfer served as controls. (**a**,**b**) Oxygen consumption rate (OCR) measured using the Seahorse XFe96 Extracellular Flux Analyzer demonstrates enhanced mitochondrial respiration in cells receiving Protocol A mitochondria compared to Protocol B and control. (**c**) Non-mitochondrial respiration is increased in cells treated with Protocol A mitochondria relative to Protocol B. (**d**) Mitochondrial calcium retention capacity assessed using Fura-4 AM fluorescence (506 nm) shows improved calcium handling in mitochondria isolated with Protocol A compared to Protocol B. (**e**) Succinate dehydrogenase (SDH) activity, measured spectrophotometrically, is elevated in mitochondria isolated using Protocol A, indicating enhanced respiratory enzyme function. (**f**,**g**) Mitochondrial reactive oxygen species (ROS) levels were assessed using Mito SOX Red. Representative confocal images (**f**) and quantification of fluorescence intensity per recipient HEK293T cell (**g**) demonstrate increased ROS production in cells receiving Protocol B PTCs mitochondria(donors). Scale bar, 20 µm. Each dot represents the mean of one independent experiment (*n* = 7 per group for ROS quantification). All other data are presented as mean ± S.E.M. (*n* = 3 independent experiments). Statistical significance was determined using an unpaired two-tailed Student’s *t*-test (ns, not significant; * *p* < 0.05, ** *p* < 0.01; **** *p* < 0.0001).

Oxygen consumption rate (OCR) was measured in HEK293T cells following transplantation with mitochondria isolated using Protocols A and B using a Seahorse XF Analyzer. Transplanted mitochondria from Protocol A exhibited significantly higher basal and maximal OCR, as well as increased non-mitochondrial respiration, compared with Protocol B (Figure 4a–c), indicating enhanced bioenergetic capacity. In addition, we performed mitochondrial transplantation experiments in cells with depleted mitochondrial DNA (mtDNA) induced by ethidium bromide treatment, as shown in Supplemental Figure S4. Despite mtDNA depletion, mitochondrial transplantation remained efficient and functionally detectable, further supporting the robustness of the approach under conditions of impaired endogenous mitochondrial activity. Supplementary Video S1 shows dynamic interactions between native mitochondria labeled in red and transplanted mitochondria labeled in green, demonstrating mitochondrial integration and network dynamics following transplantation.

Calcium retention capacity, a surrogate measure of mitochondrial membrane integrity and permeability transition, was assessed using the calcium-sensitive dye Fura-4 AM. Isolated mitochondria were incubated with Fura-4, and fluorescence was measured at 506 nm. Mitochondria from Protocol A displayed significantly greater calcium retention than those from Protocol B (Figure 4d), consistent with improved membrane integrity and functional stability.

Mitochondrial enzymatic activity was evaluated by measuring succinate dehydrogenase (SDH; Complex II) activity using a ferricyanide-based spectrophotometric assay. Isolated mitochondria (0.1 mg protein) were incubated in phosphate buffer containing potassium ferricyanide as an exogenous electron acceptor, and the reduction of ferricyanide to ferrocyanide upon addition of succinate was monitored as a decrease in absorbance at 420 nm.

Mitochondria isolated using Protocol A exhibited significantly higher SDH activity, as indicated by an increased rate of ferricyanide reduction, whereas Protocol B mitochondria showed markedly reduced activity (Figure 4e). These findings demonstrate superior preservation of mitochondrial enzymatic function with Protocol A.

Mitochondrial reactive oxygen species (ROS) production was assessed using Mito SOX Red, a mitochondrial superoxide indicator. Following transplantation into HEK293T cells, MitoSOX fluorescence was imaged by confocal microscopy and quantified in Fiji (ImageJ, 5.1i) by measuring red puncta per cell. Mitochondria from Protocol A exhibited lower ROS levels compared with Protocol B (Figure 4f,g), which may reflect reduced oxidative stress but also diminished metabolic activity and limited functional integration.

Collectively, these bioenergetic and functional analyses demonstrate that mitochondria isolated using Protocol A retain superior respiratory capacity, enzymatic activity, membrane integrity, and overall functional competence compared with those isolated using Protocol B.

### 3.5. Recovery and Functional Competence of Stored Mitochondria

**Figure 5:**
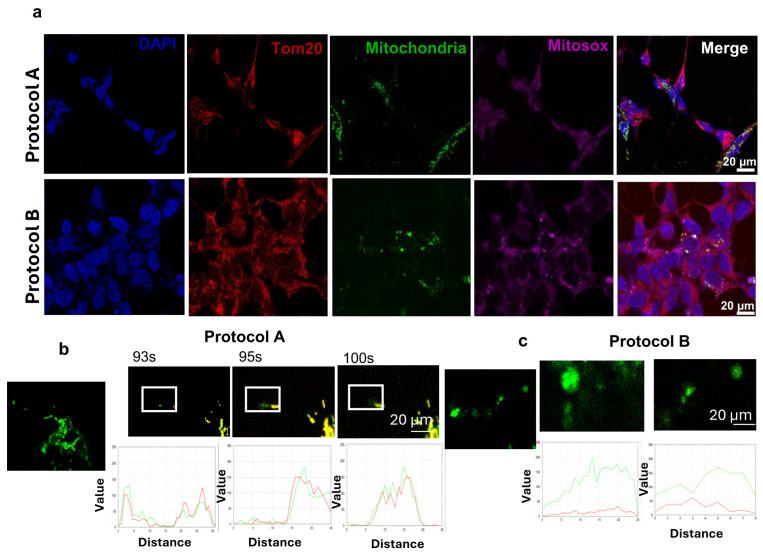
Functional assessment of cryopreserved mitochondria after transplantation. Dendra2-labeled mitochondria isolated using Protocol A or Protocol B were cryopreserved, recovered, and transferred into recipient cells. Analysis was performed at 48 h. (**a**) Immunofluorescence showing transplanted mitochondria (green), recipient mitochondrial network (TOM20, red), and mitochondrial ROS (Mito SOX, purple). Protocol A mitochondria exhibit efficient incorporation and reduced oxidative stress, whereas Protocol B mitochondria appear fragmented/rounded with increased Mito SOX signal. (**b**,**c**) Cryo-recovered mitochondrial fission-fusion dynamics assessed by Dendra2 photoconversion (405 nm) and time-lapse confocal microscopy. Line-scan analysis (green and red lines) across fusion interfaces demonstrates redistribution of red signal into adjacent green mitochondria, indicating matrix content exchange. Protocol B mitochondria display limited fusion and reduced network dynamics.

To evaluate the therapeutic potential of stored mitochondria, mitochondria were isolated using Protocols A and B, preserved in a DMSO-based recovery buffer at −80 °C for ≥3 days and assessed following transplantation (Figure 5a). Functional recovery and mitochondrial dynamics were evaluated 48 h post-transplantation.

Recipient cell mitochondria were labeled with Mito Tracker Red, whereas transplanted mitochondria were identified by green fluorescence. To assess mitochondrial oxidative status, Mito SOX staining (purple) was performed (Supplementary Video S1). Mitochondria recovered from Protocol A were efficiently internalized, exhibited preserved morphology, and integrated with the endogenous mitochondrial network. In contrast, mitochondria isolated using Protocol B showed increased oxidative stress and were associated with impaired cellular mitochondrial organization.

Mitochondrial fusion was further assessed using the photoconvertible Dendra2 system. The photoconversion-based FRAP analysis revealed active fusion events in HEK293T (Supplementary Video S2).

Cells transplanted with cryopreserved mitochondria from Protocol A (Figure 5b), exhibited functional integration and dynamic network remodeling. In contrast, mitochondria from Protocol B displayed minimal dynamic behavior and failed to exhibit fusion following storage and recovery. The quantitative analysis confirmed that only mitochondria from Protocol A retained fusion capacity after cryopreservation (Figure 5b, lower panels).

Collectively, these findings demonstrate that mitochondria isolated using Protocol A preserve structural integrity, dynamic activity (Supplementary Video S3), and fusion competence following DMSO-based storage, highlighting their superior potential for therapeutic mitochondrial transplantation.

## 4. Discussion

This study provides a comprehensive comparison of two mitochondrial isolation strategies, emphasizing not only biochemical purity but, critically, post-transfer functionality. Our findings demonstrate that preservation of mitochondrial integrity, dynamics, and bioenergetic capacity is highly dependent on the isolation approach. We establish a rapid, detergent-free, differential centrifugation-based method (Protocol A) that maintains mitochondrial viability and supports efficient transplantation.

A central strength of our approach is the use of mitochondria-targeted Dendra2-expressing cells, which enable direct, real-time assessment of mitochondrial behavior following transfer. As previously described, Dendra2 is a photoconvertible fluorescent protein that irreversibly switches from green to red upon 405 nm illumination, allowing selective labeling of a subpopulation of mitochondria and tracking of their fate over time [26,27,28]. When targeted to the mitochondrial matrix, this system permits high-resolution analysis of mitochondrial dynamics, including fusion, transport, and network remodeling in live cells. Importantly, fusion events can be directly quantified by the mixing of photoconverted (red) and non-converted (green) mitochondrial signals, reflecting matrix continuity and functional integration. This approach provides a robust and sensitive readout of mitochondrial functionality that extends beyond static morphological or biochemical measurements.

Across all functional readouts, Protocol A consistently outperformed Protocol B, reflecting superior preservation of mitochondrial bioenergetics and structural integrity. Mitochondria isolated using Protocol A exhibited higher oxygen consumption rates (OCR), which directly reflect electron transport chain (ETC) activity and oxidative phosphorylation capacity. OCR is a primary indicator of mitochondrial respiration, and reductions in OCR are widely associated with impaired mitochondrial function and energy production. The elevated OCR observed with Protocol A, therefore, indicates intact respiratory chain activity and efficient ATP-generating capacity following transplantation.

Consistent with this, mitochondria isolated using Protocol A displayed increased succinate dehydrogenase (SDH; Complex II) activity, a key enzyme linking the tricarboxylic acid (TCA) cycle to the ETC. SDH activity serves as a proxy for mitochondrial metabolic competence, as it reflects both substrate oxidation and electron transfer into the respiratory chain. Higher SDH activity suggests preservation of enzymatic integrity and mitochondrial matrix function, which are often compromised during harsh isolation procedures [29].

In parallel, Protocol A mitochondria demonstrated enhanced calcium retention capacity, a sensitive indicator of mitochondrial membrane potential and resistance to permeability transition pore (mPTP) opening. Calcium retention reflects the ability of mitochondria to buffer Ca^2+^ and maintain inner membrane integrity before a catastrophic permeability transition occurs. Increased calcium retention in Protocol A mitochondria indicates preserved membrane polarization and reduced susceptibility to stress-induced dysfunction, both of which are essential for maintaining ATP production and cell survival [27].

Morphological and network-level observations further supported these functional advantages. Mitochondria isolated using Protocol A retained elongated, interconnected structures and exhibited active fusion dynamics, whereas Protocol B mitochondria appeared fragmented and rounded. Mitochondrial morphology is tightly coupled to function: elongated, fused networks are associated with efficient oxidative metabolism and stress resistance, while fragmentation is linked to bioenergetic failure and impaired mitochondrial quality control. Together, these data demonstrate that Protocol A preserves not only biochemical activity but also the structural and dynamic properties required for functional mitochondrial integration. These mitochondria exhibited elongated, interconnected structures, robust fusion dynamics, and efficient integration into recipient cells, as demonstrated by Dendra2-based photoconversion assays. In contrast, mitochondria isolated using Protocol B, despite higher apparent purity, were predominantly rounded, lacked fusion and fission activity, and showed minimal uptake and integration following transplantation. These findings indicate that structural and functional competence, not purity alone, is the primary determinant of mitochondrial performance in transplantation contexts.

A key observation is that rapid isolation and avoidance of harsh chemical or detergent-based steps in Protocol A likely preserve mitochondrial membrane integrity, protein complexes, and cytoskeletal interactions necessary for dynamic remodeling. This is reflected in the enhanced fusion events, network formation, and sustained bioenergetic activity observed post-transfer. Conversely, the commercial kit-based Protocol B, while effective for generating highly purified mitochondrial fractions (as evidenced by minimal GAPDH and LAMP2 contamination), appears to compromise mitochondrial plasticity and functional recovery, limiting its applicability for dynamic or therapeutic studies.

Importantly, we demonstrate that mitochondria isolated using Protocol A retain structural integrity, bioenergetic activity, and fusion competence following cryopreservation in a DMSO-based recovery buffer. After thawing, these mitochondria remained capable of integrating into recipient cells, exhibited preserved mitochondrial dynamics, and maintained functional respiratory activity, indicating that the isolation procedure itself is a critical determinant of successful post-thaw recovery. These findings support the feasibility of establishing standardized mitochondrial storage strategies and provide proof-of-concept for the development of mitochondrial biobanks capable of supplying ready-to-use organelles for experimental and, potentially, therapeutic applications.

The concept of mitochondrial biobanking offers several potential advantages over the current practice of isolating fresh mitochondria immediately before each experiment or clinical procedure. Standardized cryopreserved mitochondrial preparations could reduce inter-experimental variability, facilitate multicenter studies, simplify logistics for mitochondrial transplantation, and enable centralized quality-controlled manufacturing. Such an approach may ultimately allow mitochondrial preparations to be generated from carefully characterized donor sources, stored under standardized conditions, and distributed on demand in a manner analogous to current blood, stem-cell, or tissue biobanking systems.

Despite these encouraging findings, several important challenges remain before clinical implementation becomes feasible. First, the present study evaluated relatively short-term storage, whereas the effects of prolonged cryostorage over weeks, months, or years remain unknown. Extended storage may progressively impair mitochondrial membrane integrity, respiratory chain activity, mitochondrial DNA stability, and the dynamic fusion–fission machinery required for successful functional integration after transplantation. Future studies should therefore systematically define storage duration limits and establish acceptable functional thresholds for clinical-grade mitochondrial preparations.

Second, standardized quality-control criteria will be essential before cryopreserved mitochondria can be used therapeutically. While ATP production and oxygen consumption are frequently used as measures of mitochondrial function, additional release criteria should include preservation of mitochondrial membrane potential, respiratory control ratio, calcium retention capacity, mitochondrial ROS production, structural integrity assessed by electron microscopy, mitochondrial DNA integrity and copy number, and the ability to undergo fusion and integrate into recipient mitochondrial networks after transplantation. Together, these parameters would provide a comprehensive assessment of mitochondrial viability beyond simple biochemical purity.

Another challenge concerns manufacturing reproducibility and scalability. Mitochondrial yield and functional competence are influenced by donor cell type, metabolic state, tissue source, donor age, disease status, isolation timing, and handling procedures. Establishing standardized operating procedures that minimize batch-to-batch variability while maintaining mitochondrial functionality will be essential for large-scale production. In addition, scalable manufacturing processes compatible with Good Manufacturing Practice (GMP) requirements will need to be developed to enable widespread clinical translation.

Regulatory considerations also deserve attention. Unlike conventional biologics or cell therapies, isolated mitochondria represent a unique organelle-based therapeutic product that currently lacks a well-defined regulatory framework. Future clinical translation will require rigorous evaluation of sterility, endotoxin contamination, donor screening, traceability, product stability, potency assays, and batch release criteria. Furthermore, issues related to allogeneic versus autologous mitochondrial sources, immunological compatibility, and long-term biosafety remain incompletely understood and warrant careful investigation before routine clinical application.

Our findings should also be interpreted in the context of previous cryopreservation studies. Earlier reports have demonstrated preservation of isolated mitochondria using cryoprotectants such as glycerol combined with sucrose [27], primarily for biochemical characterization after thawing. However, these studies generally focused on maintaining respiratory activity or enzymatic function and did not evaluate the ability of stored mitochondria to be internalized by recipient cells or to restore mitochondrial dynamics following transplantation. In contrast, our protocol combines rapid detergent-free isolation with DMSO-based cryopreservation and demonstrates preservation of multiple functional endpoints relevant to mitochondrial transplantation, including cellular uptake, respiratory activity, mitochondrial network remodeling, and fusion competence after thawing. Nevertheless, direct comparisons among different cryoprotective formulations, including glycerol-, trehalose-, polyethylene glycol-, and DMSO-based preservation strategies, together with optimization of cooling rates, storage temperatures, thawing conditions, and cryoprotectant concentrations, will be necessary to determine the most effective approach for long-term mitochondrial preservation.

Overall, our findings establish an important proof-of-concept that functional mitochondria can be isolated rapidly, cryopreserved, and recovered while maintaining key characteristics required for transplantation. Although additional work is needed to optimize long-term preservation, establish standardized quality-control metrics, reduce manufacturing variability, and address regulatory requirements, the present protocol provides an important foundation toward the development of clinically applicable mitochondrial biobanks and organelle-based therapeutics.

Collectively, our results reveal a fundamental trade-off between mitochondrial purity and functional viability. While highly purified preparations may be advantageous for biochemical or signaling assays, they do not necessarily preserve essential physiological properties such as fusion, fission, and metabolic activity. These findings underscore the importance of optimizing isolation conditions, including buffer composition, mechanical handling, processing time, and cryopreservation strategy, to maintain mitochondrial integrity. From a translational perspective, Protocol A provides a robust platform for mitochondrial transplantation and establishes a framework for advancing organelle-based therapies.

In summary, interest in mitochondrial transplantation therapy has grown substantially in recent years, with studies demonstrating its potential to restore kidney function [28], improve cardiac recovery [30], and treat mitochondria-related disorders [31].

## 5. Patents

U.S. Provisional Patent Application No. 63/876,191, Title: “DEVELOPMENT OF AN EFFECTIVE PROTOCOL FOR ISOLATING METABOLICALLY ACTIVE MITOCHONDRIA FOR USE IN ORGANELLE-BASED THERAPY (MITOCHONDRIAL TRANSPLANTATION)”. Inventor: Rihab Bouchareb. Temple Ref.: C2025-056 & C2025-067 (Bouchareb). Our Ref.: 206017-0329-P1US.

## Data Availability

The original contributions presented in this study are included in the article/Supplementary Materials. Further inquiries can be directed to the corresponding author.

## Supplementary Materials

The following supporting information can be downloaded at: https://www.mdpi.com/article/10.3390/cells15141279/s1, Figure S1: Analysis of cell survival of Primary proximal tubular cells (PTCs) in vitro. PTCs were cultured in DMEM/F-12 medium supplemented with 5% fetal bovine serum (FBS) until reaching 70–80% confluency. On the day of treatment, the culture medium was replaced, and cells were exposed to hydrogen peroxide (H_2_O_2_) at a final concentration of 600 µM for 18 h. Apoptotic cells (Annexin V–positive) were quantified using ImageJ 5.1i software and normalized to the total number of cells. Statistical significance was determined using an unpaired *t*-test in GraphPad Prism, with results expressed as mean ± SEM. Figure S2a: Gating strategy using flow cytometry to assess the efficiency of mitochondria transplantation from protocols A and B in HEK cells using green, fluorescent mitochondria; Figure S2b: Gating strategy using flow cytometry to assess the efficiency of mitochondria transplantation from protocols A and B in HEK cells using green fluorescent mitochondria; Figure S3: HEK293 cell oxygen consumption rate (OCR). HEK293 cells were maintained in complete culture medium supplemented with 1% penicillin–streptomycin before OCR analysis; Figure S4: Original pictures of gels used for western blotting; Figure S5: Transplantation of primary cell culture of mouse heart fibroblasts (recipient) with mitochondria using protocols A (right) and B (left). Black arrows pointing to the transplanted green fluorescent mitochondria. Video S1: Phase-contrast and fluorescence live-cell imaging illustrating the intracellular integration of transplanted mitochondria (green) within recipient cells, alongside native mitochondrial networks (red). Video S2: Original Video used for FRAP quantification in Figure 3b-c. Video S3: Original Video used for FRAP quantification in Figure 5b, illustrating the assessment of the mitochondrial fusion used in Figure 5b.

## Abbreviations

The following abbreviations are used in this manuscript:

OCR	oxygen consumption rate
SDH	succinate dehydrogenase (SDH) activity

## References

[1] Moura J.P., Oliveira P.J., Urbano A.M. (2025). Mitochondria: An overview of their origin, genome, architecture, and dynamics. Biochim. Biophys. Acta Mol. Basis Dis..

[2] Zong Y., Li H., Liao P., Chen L., Pan Y., Zheng Y., Zhang C., Liu D., Zheng M., Gao J. (2024). Mitochondrial dysfunction: Mechanisms and advances in therapy. Signal Transduct. Target. Ther..

[3] Martinez-Reyes I., Chandel N.S. (2020). Mitochondrial TCA cycle metabolites control physiology and disease. Nat. Commun..

[4] Arnold P.K., Finley L.W.S. (2023). Regulation and function of the mammalian tricarboxylic acid cycle. J. Biol. Chem..

[5] Jimenez-Uribe A.P., Hernandez-Cruz E.Y., Ramirez-Magana K.J., Pedraza-Chaverri J. (2021). Involvement of Tricarboxylic Acid Cycle Metabolites in Kidney Diseases. Biomolecules.

[6] Chandel N.S. (2021). Nucleotide Metabolism. Cold Spring Harb. Perspect. Biol..

[7] Ciccarone F., Vegliante R., Di Leo L., Ciriolo M.R. (2017). The TCA cycle as a bridge between oncometabolism and DNA transactions in cancer. Semin. Cancer Biol..

[8] Sanchez-Garcia F.J., Perez-Hernandez C.A., Rodriguez-Murillo M., Moreno-Altamirano M.M.B. (2021). The Role of Tricarboxylic Acid Cycle Metabolites in Viral Infections. Front. Cell. Infect. Microbiol..

[9] Chandel N.S. (2021). Amino Acid Metabolism. Cold Spring Harb. Perspect. Biol..

[10] Guo D., He H., Meng Y., Luo S., Lu Z. (2023). Determiners of cell fates: The tricarboxylic acid cycle versus the citrate-malate shuttle. Protein Cell.

[11] Glover H.L., Schreiner A., Dewson G., Tait S.W.G. (2024). Mitochondria and cell death. Nat. Cell Biol..

[12] Rambold A.S., Lippincott-Schwartz J. (2011). Mechanisms of mitochondria and autophagy crosstalk. Cell Cycle.

[13] Murphy M.P. (2009). How mitochondria produce reactive oxygen species. Biochem. J..

[14] Zhou B., Tian R. (2018). Mitochondrial dysfunction in pathophysiology of heart failure. J. Clin. Investig..

[15] Hoogstraten C.A., Hoenderop J.G., de Baaij J.H.F. (2024). Mitochondrial Dysfunction in Kidney Tubulopathies. Annu. Rev. Physiol..

[16] Klemmensen M.M., Borrowman S.H., Pearce C., Pyles B., Chandra B. (2024). Mitochondrial dysfunction in neurodegenerative disorders. Neurotherapeutics.

[17] Prasun P. (2020). Mitochondrial dysfunction in metabolic syndrome. Biochim. Biophys. Acta Mol. Basis Dis..

[18] Hsu C.C., Tseng L.M., Lee H.C. (2016). Role of mitochondrial dysfunction in cancer progression. Exp. Biol. Med..

[19] Luo H., Lai Y., Tang W., Wang G., Shen J., Liu H. (2024). Mitochondrial transplantation: A promising strategy for treating degenerative joint diseases. J. Transl. Med..

[20] Lee A.R., Woo J.S., Lee S.Y., Na H.S., Cho K.H., Lee Y.S., Lee J.S., Kim S.A., Park S.H., Kim S.J. (2022). Mitochondrial Transplantation Ameliorates the Development and Progression of Osteoarthritis. Immune Netw..

[21] Wang X., Liu Z., Zhang L., Hu G., Tao L., Zhang F. (2024). Mitochondrial transplantation for the treatment of cardiac and noncardiac diseases: Mechanisms, prospective, and challenges. Life Med..

[22] Liao P.C., Bergamini C., Fato R., Pon L.A., Pallotti F. (2020). Isolation of mitochondria from cells and tissues. Methods Cell Biol..

[23] Ngo J., Benador I.Y., Brownstein A.J., Vergnes L., Veliova M., Shum M., Acin-Perez R., Reue K., Shirihai O.S., Liesa M. (2021). Isolation and functional analysis of peridroplet mitochondria from murine brown adipose tissue. STAR Protoc..

[24] Williamson C.D., Wong D.S., Bozidis P., Zhang A., Colberg-Poley A.M. (2015). Isolation of Endoplasmic Reticulum, Mitochondria, and Mitochondria-Associated Membrane and Detergent Resistant Membrane Fractions from Transfected Cells and from Human Cytomegalovirus-Infected Primary Fibroblasts. Curr. Protoc. Cell Biol..

[25] Picard M., Taivassalo T., Gouspillou G., Hepple R.T. (2011). Mitochondria: Isolation, structure and function. J. Physiol..

[26] Awasthi V., Sharief S.Q., Bkhache M., Das A., Bouchareb R. (2025). Assessment of Mitochondrial Fission/Fusion Dynamics in Kidney Proximal Tubular Cells. J. Vis. Exp..

[27] Bouchareb R., Yu L., Lassen E., Daehn I.S. (2022). Isolation of Conditionally Immortalized Mouse Glomerular Endothelial Cells with Fluorescent Mitochondria. J. Vis. Exp..

[28] Pham A.H., McCaffery J.M., Chan D.C. (2012). Mouse lines with photo-activatable mitochondria to study mitochondrial dynamics. Genesis.

[29] Franko A., Baris O.R., Bergschneider E., von Toerne C., Hauck S.M., Aichler M., Walch A.K., Wurst W., Wiesner R.J., Johnston I.C. (2013). Efficient isolation of pure and functional mitochondria from mouse tissues using automated tissue disruption and enrichment with anti-TOM22 magnetic beads. PLoS ONE.

[30] Ahmad F., Alamoudi W., Haque S., Salahuddin M., Alsamman K. (2018). Simple, reliable, and time-efficient colorimetric method for the assessment of mitochondrial function and toxicity. Biomol. Biomed..

[31] Jang S., Chapa-Dubocq X.R., Fossati S., Javadov S. (2021). Analysis of Mitochondrial Calcium Retention Capacity in Cultured Cells: Permeabilized Cells Versus Isolated Mitochondria. Front. Physiol..

