# Supplementary material for: Optimized Isolation and Cryopreservation of Functional Mitochondria for Transplantation and Therapeutic Applications
Source: Cells. 2026 Jul 16;15(14):1279. doi: 10.3390/cells15141279 (PMC13406654; doi:10.3390/cells15141279)
Supplement: Supplementary file 1 [file cells-15-01279-s001.zip › cells-4403387-fine-supplementary-7.15/cells-4403387-fine-supplement Figures resubmission.pptx]

## Slide 1
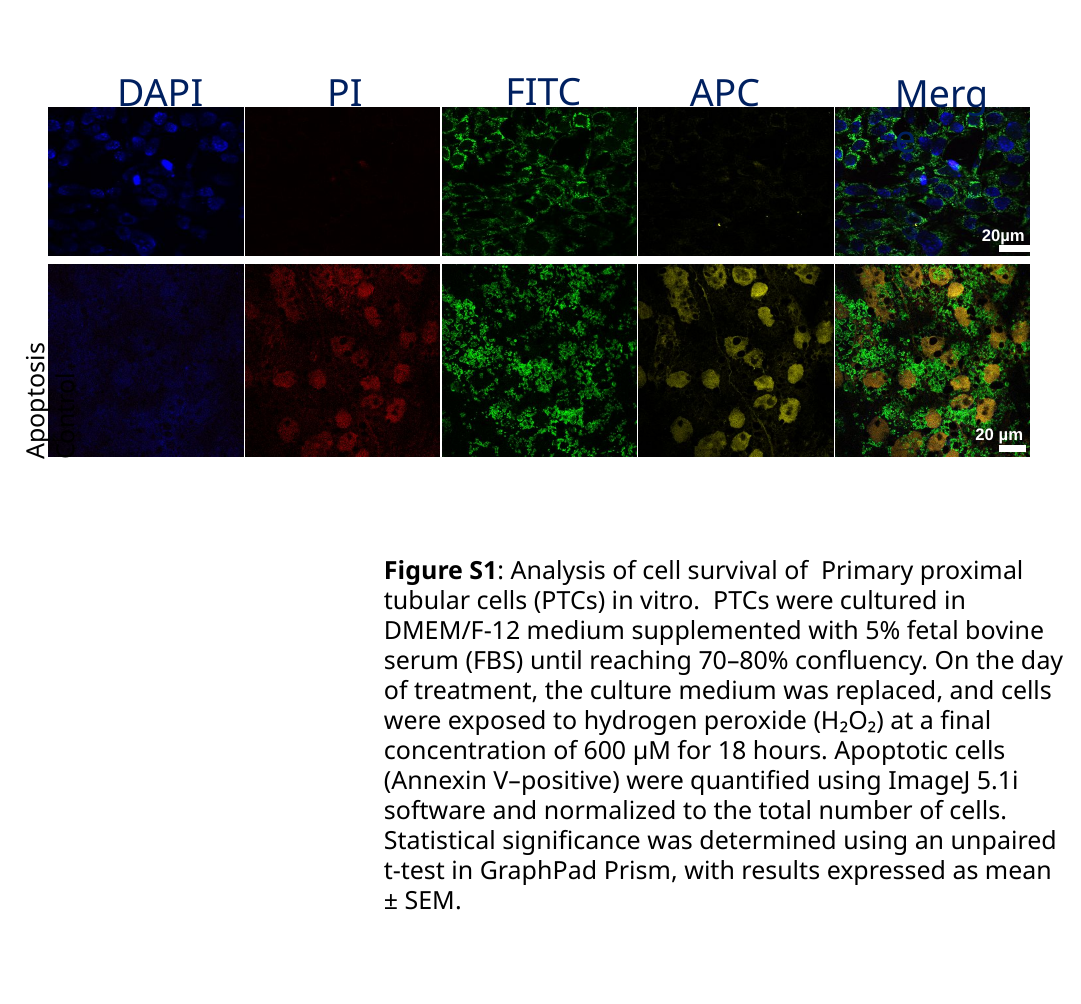

FITC
DAPI
PI
APC
Merge
20µm
20 µm
Apoptosis Control
Figure S1: Analysis of cell survival of Primary proximal tubular cells (PTCs) in vitro. PTCs were cultured in DMEM/F-12 medium supplemented with 5% fetal bovine serum (FBS) until reaching 70–80% confluency. On the day of treatment, the culture medium was replaced, and cells were exposed to hydrogen peroxide (H₂O₂) at a final concentration of 600 µM for 18 hours. Apoptotic cells (Annexin V–positive) were quantified using ImageJ 5.1i software and normalized to the total number of cells.
Statistical significance was determined using an unpaired t-test in GraphPad Prism, with results expressed as mean ± SEM.

## Slide 2
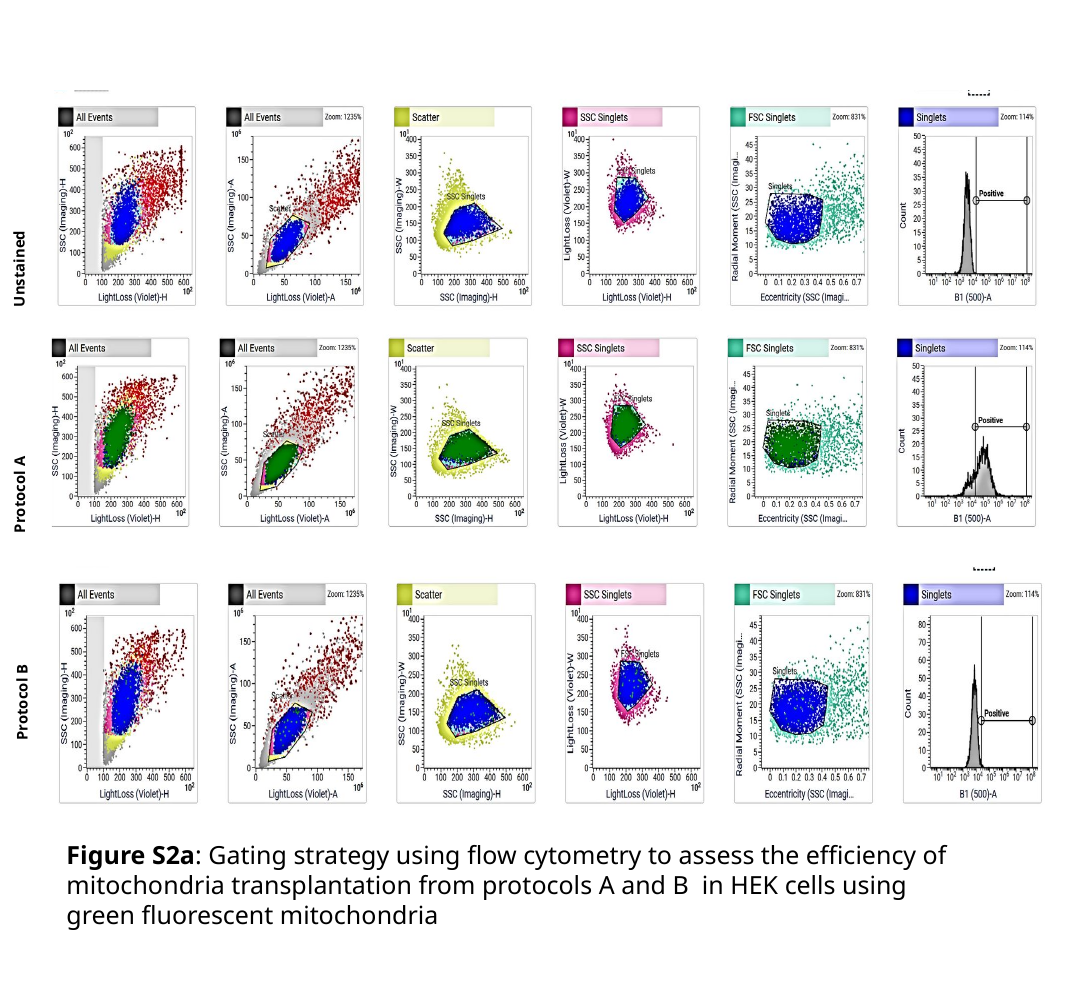

Unstained
Protocol A
Protocol B
Figure S2a: Gating strategy using flow cytometry to assess the efficiency of mitochondria transplantation from protocols A and B in HEK cells using green fluorescent mitochondria

## Slide 3
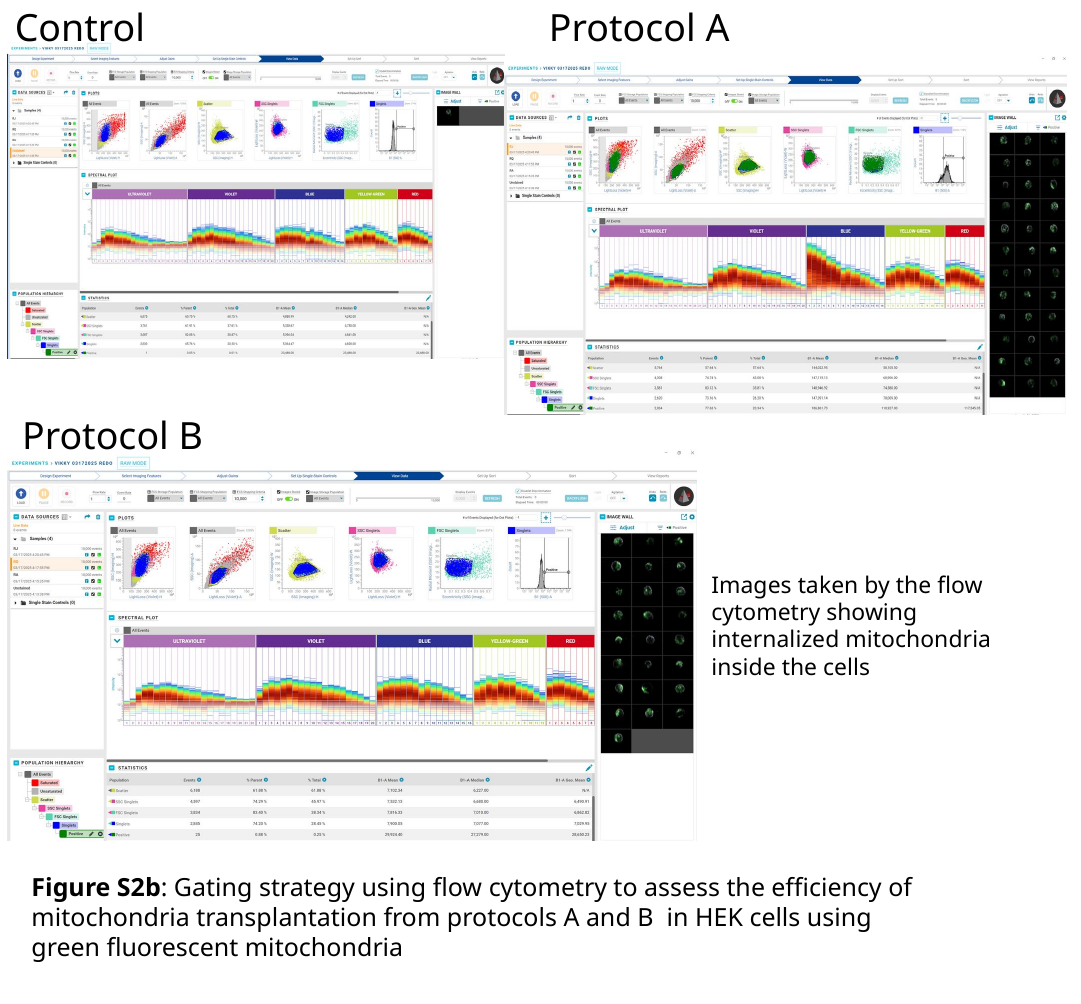

Control
Protocol A
Protocol B
Images taken by the flow cytometry showing internalized mitochondria inside the cells
Figure S2b: Gating strategy using flow cytometry to assess the efficiency of mitochondria transplantation from protocols A and B in HEK cells using green fluorescent mitochondria

## Slide 4
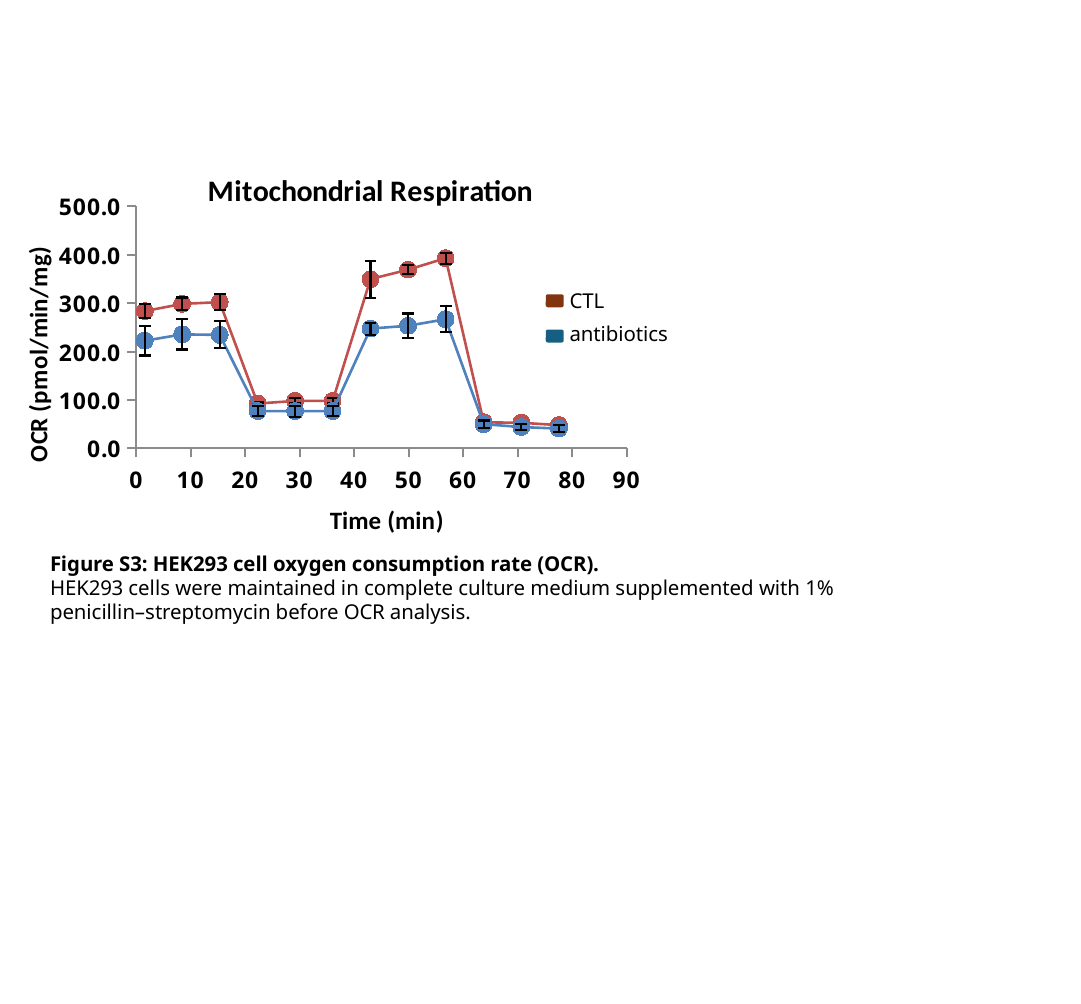

### Chart: Mitochondrial Respiration
| Category | FEMALE TUBULAR CELL CTRL | FEMALE TUBULAR CELL MITO TRANSPLANT J | Unassigned | Unselected |
|---|---|---|---|---|CTL
antibiotics
Figure S3: HEK293 cell oxygen consumption rate (OCR).
HEK293 cells were maintained in complete culture medium supplemented with 1% penicillin–streptomycin before OCR analysis.

## Slide 5
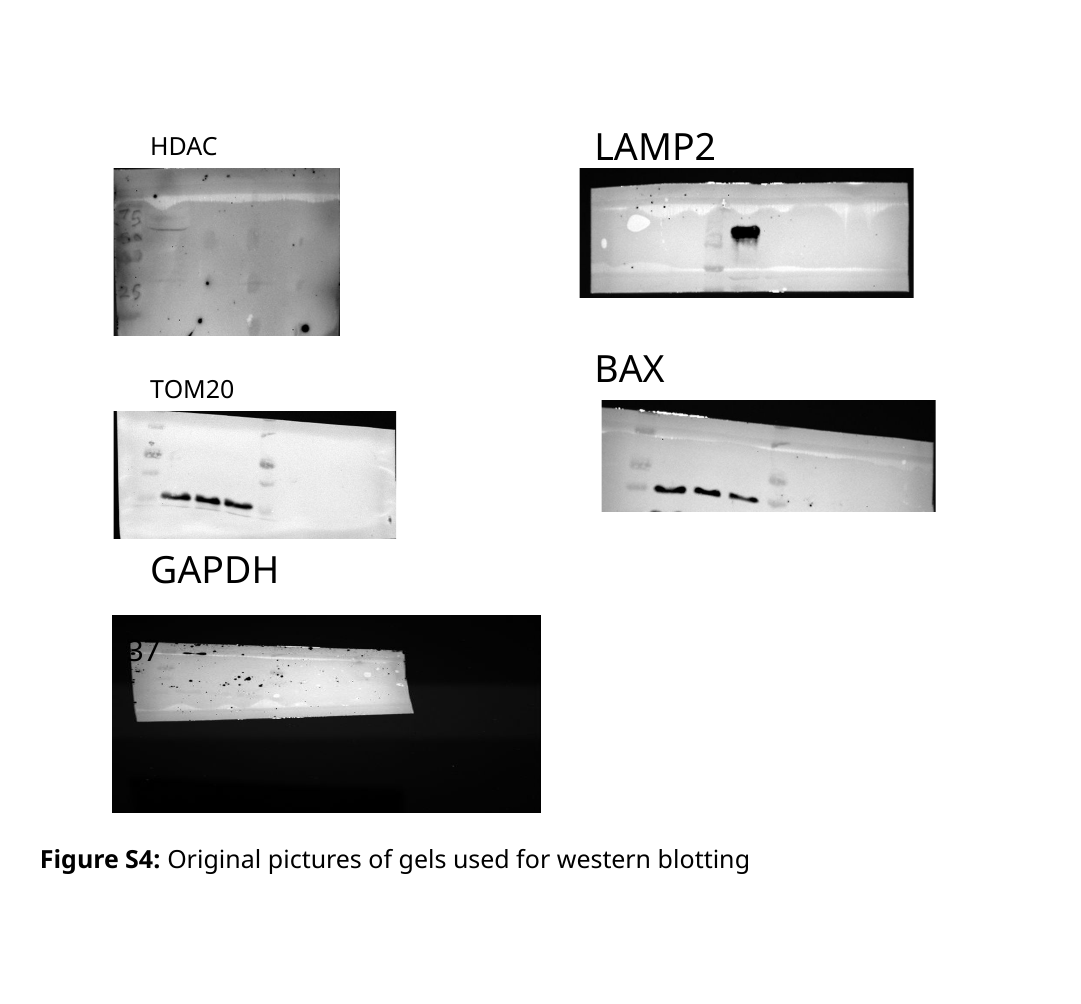

LAMP2
HDAC
BAX
TOM20
GAPDH
37
Figure S4: Original pictures of gels used for western blotting

## Slide 6
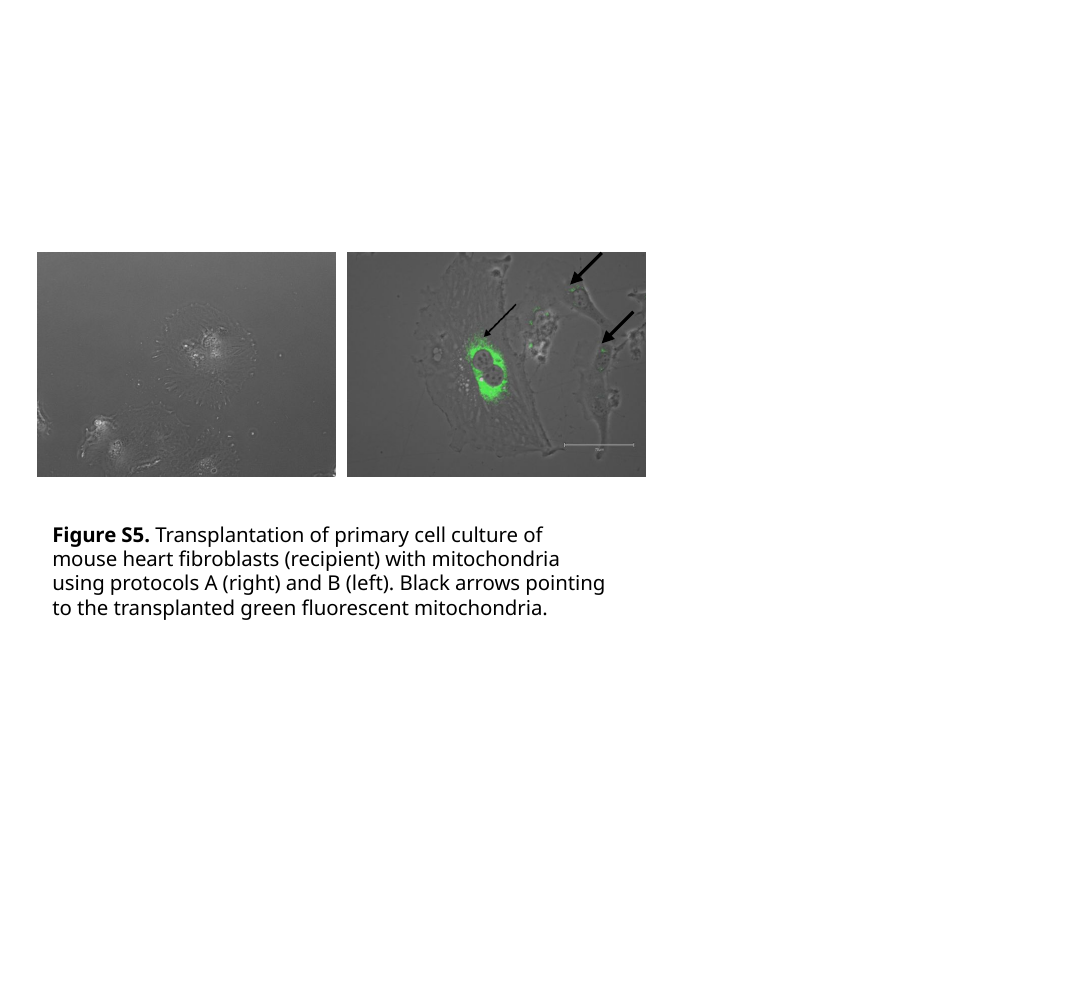

Figure S5. Transplantation of primary cell culture of mouse heart fibroblasts (recipient) with mitochondria using protocols A (right) and B (left). Black arrows pointing to the transplanted green fluorescent mitochondria.

## Slide 7
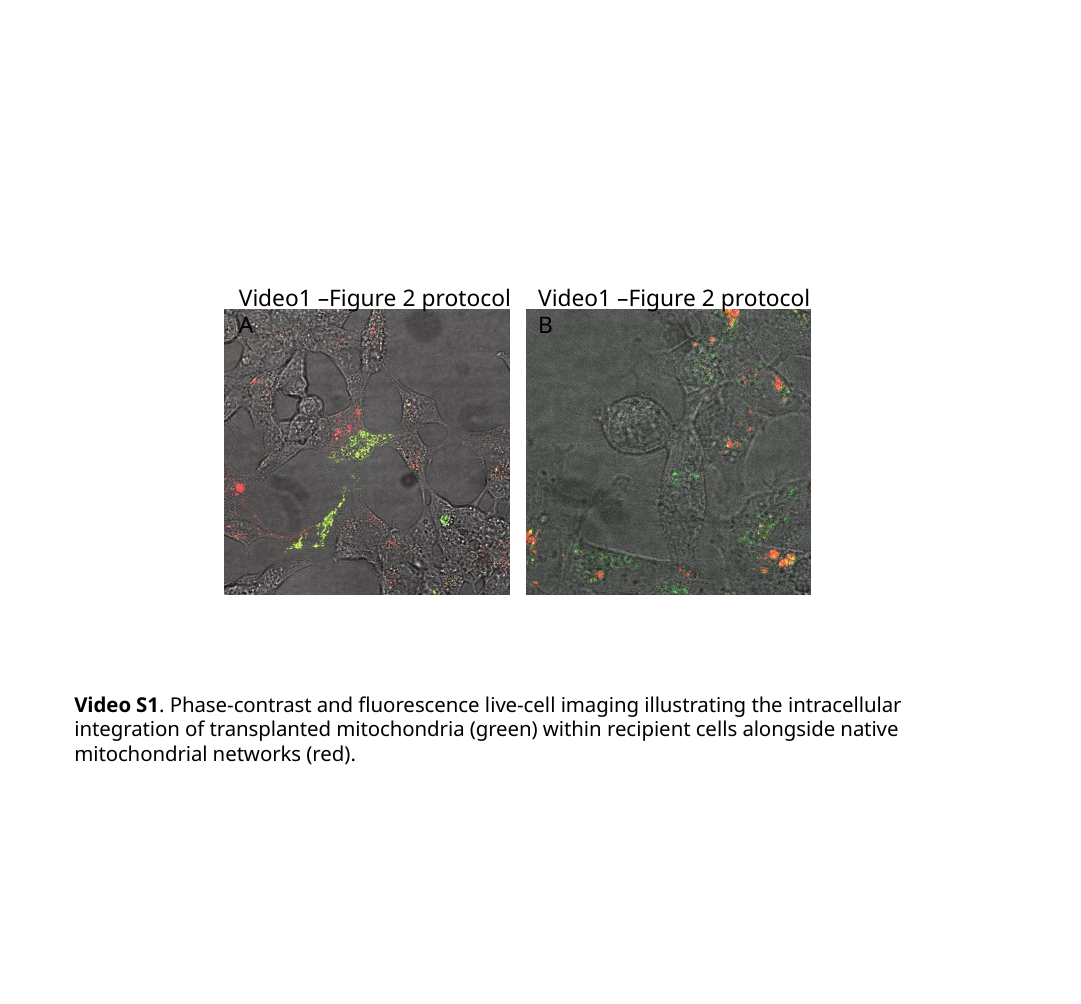

Video1 –Figure 2 protocol A
Video1 –Figure 2 protocol B
Video S1. Phase-contrast and fluorescence live-cell imaging illustrating the intracellular integration of transplanted mitochondria (green) within recipient cells alongside native mitochondrial networks (red).

## Slide 8
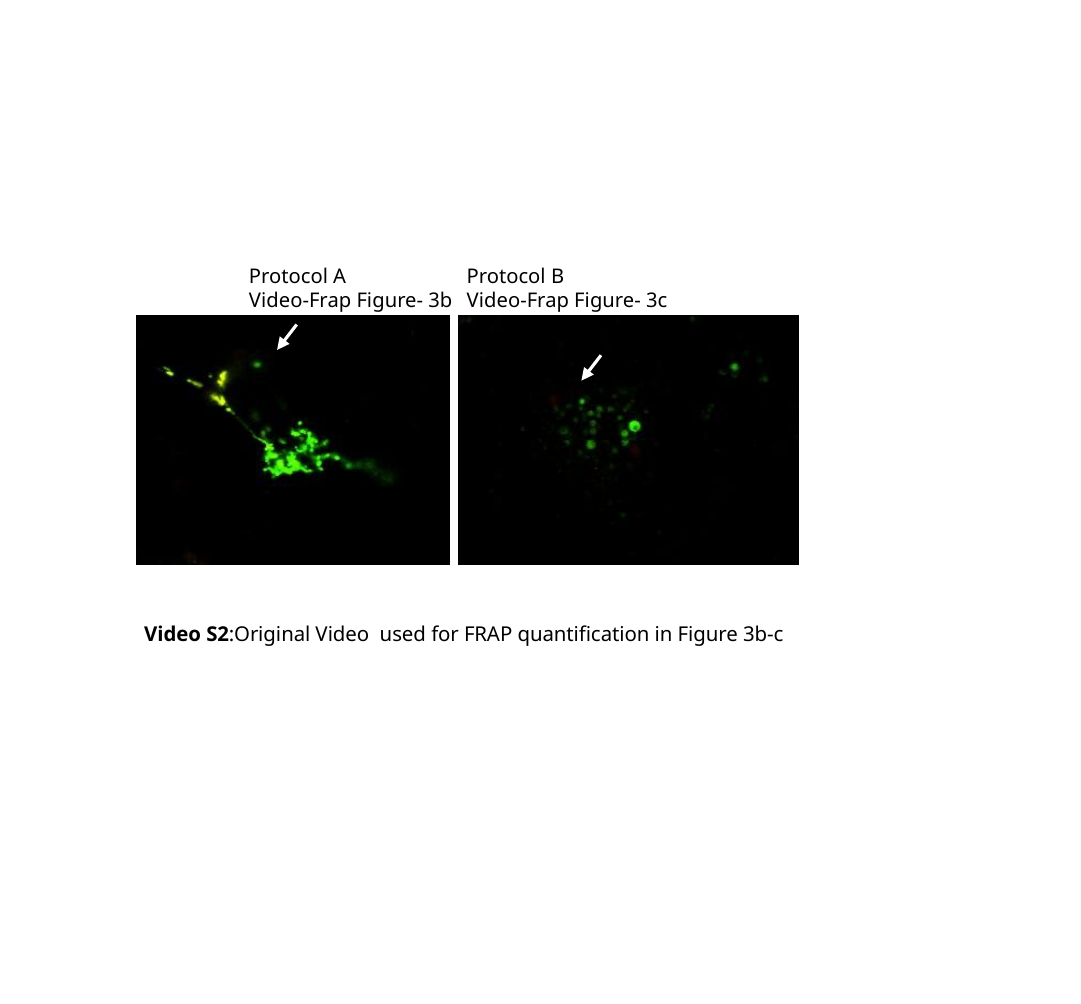

Protocol A
Video-Frap Figure- 3b
Protocol B
Video-Frap Figure- 3c
Video S2:Original Video used for FRAP quantification in Figure 3b-c

## Slide 9
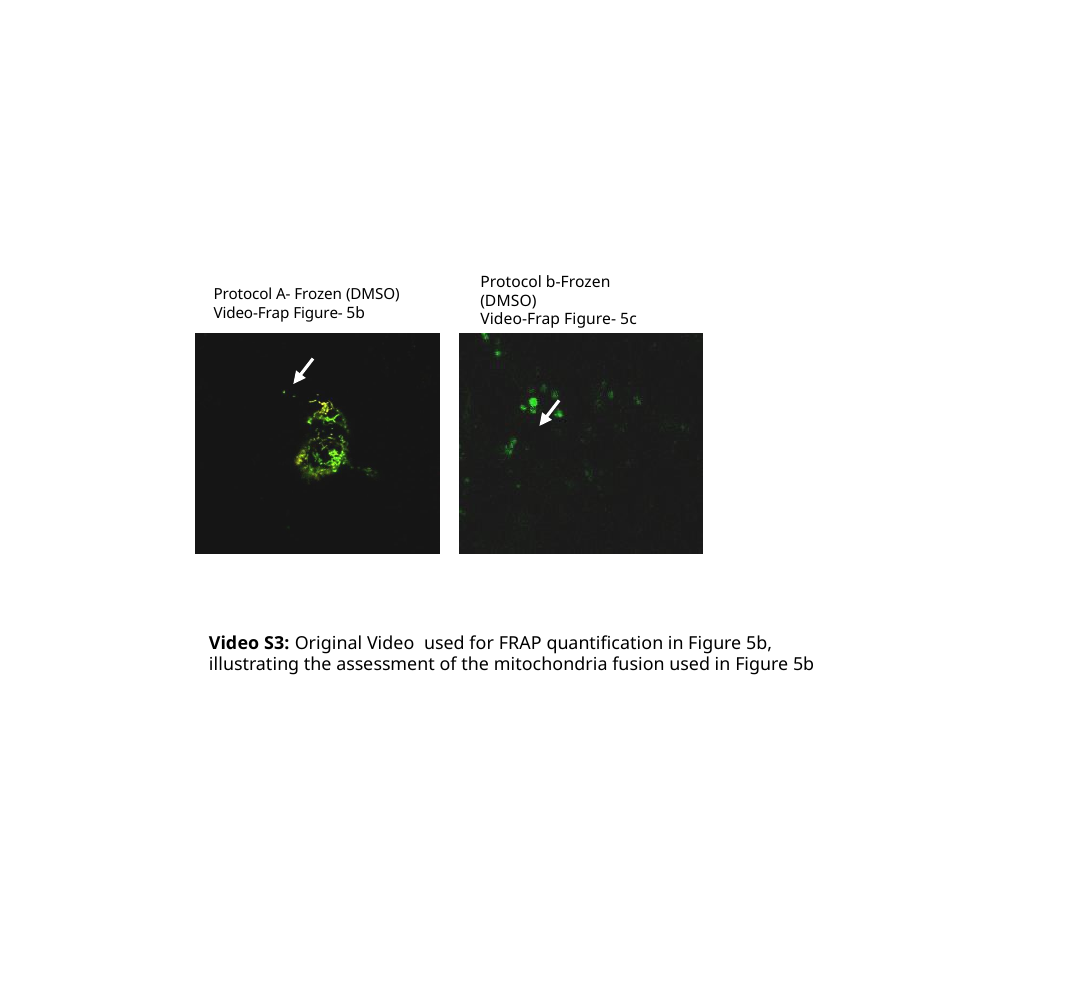

Protocol b-Frozen (DMSO)
Video-Frap Figure- 5c
Protocol A- Frozen (DMSO)
Video-Frap Figure- 5b
Video S3: Original Video used for FRAP quantification in Figure 5b, illustrating the assessment of the mitochondria fusion used in Figure 5b
